# Understanding Current Trends and Advances in Transarterial Radioembolization Dosimetry

**DOI:** 10.3390/diagnostics16010043

**Published:** 2025-12-23

**Authors:** Shamar Young, Kiyon Naser-Tavakolian, Abin Sajan, Stephen Reis, Gregory Woodhead, Tyler Sandow, Juan Gimenez, Kirema Garcia-Reyes, Zachary Berman, Venkatesh P. Krishnasamy

**Affiliations:** 1 Interventional Radiology, University of Arizona Tucson, Tucson, AZ 85724, USA; 2 Vascular and Interventional Radiology, Columbia University, New York, NY 10032, USA; 3 Interventional Radiology, Ocshner Medical Center, New Orleans, LA 70121, USA; 4 Interventional Radiology, Icahn School of Medicine at Mount Sinai, New York, NY 10029, USA; 5 Interventional Radiology, University of California San Diego, San Diego, CA 92093, USA; 6 Interventional Radiology, University of Alabama at Birmingham, Birmingham, AL 35294, USA; vkpk27@gmail.com

**Keywords:** transarterial radioembolization, Yttrium-90, voxel-based dosimetry, personalized dosimetry, liver malignancy

## Abstract

Transarterial radioembolization (TARE) is an established therapy for primary and secondary hepatic malignancies. Outcomes depend heavily on dosimetry, which has evolved from empirical and body-surface-area methods to partition and voxel-based approaches. This review summarizes current evidence for advanced (personalized) dosimetry across tumor types, highlights emerging dose–response concepts, and outlines practical barriers and implementation strategies. A narrative review of peer-reviewed clinical studies and trials evaluating dosimetry in TARE, with emphasis on hepatocellular carcinoma (HCC), intrahepatic cholangiocarcinoma (iCCA), metastatic colorectal cancer (mCRC), neuroendocrine tumor (NET), and breast cancer liver metastases, was performed with comparison of single-compartment medical internal radiation dosimetry method (MIRD), partition (multicompartment) methods, and voxel-based dosimetry methodologies. Personalized dosimetry improves outcomes in multiple tumor types. A randomized trial in HCC showed superior overall survival with partition-based dosing versus MIRD. In selective HCC treatments, voxel-derived metrics (e.g., D95) correlate with complete pathologic necrosis, suggesting benefit beyond mean dose targets. For iCCA, data associate higher tumor doses with better radiologic response, progression-free survival, and downstaging. In mCRC, voxel-based and threshold analyses link specific tumor and margin doses with metabolic/radiographic response and survival. Smaller series in NET and breast cancer indicate dose–response relationships using advanced dosimetry. Evidence supports broader adoption of advanced dosimetry in TARE. Emerging strategies that ensure adequate coverage of the “coldest” tumor regions and thoughtful particle-load planning may further optimize results. Standardized protocols, prospective validation, and scalable workflows are needed to accelerate implementation.

## 1. Introduction

Transarterial radioembolization (TARE) has become a well-established treatment method for primary and secondary malignancies of the liver [1,2,3,4,5,6]. TARE, a brachytherapy, delivers radiation attached to/embedded within minimally or non-embolic microspheres, through the arteries of the liver. As such, TARE provides several advantages, including utilization of preferential blood flow to the tumor over the adjacent normal liver tissue, allowing therapeutic doses to the tumor with limited liver toxicity. However, heterogeneous perfusion within the tumor remains a challenge. Unsurprisingly, dosimetry, the science of understanding dose distribution and thresholds, has become increasingly established as a vital component that drives TARE outcomes and overall survival [1,4,5].

Dosimetric techniques have advanced significantly over the past several decades with older non-personalized methods (i.e., empirical, body surface area (BSA), and modified body surface area (mBSA)) being replaced with more personalized techniques [7]. The ability to perform partition or voxel-based dosimetry, often referred to as advanced or personalized dosimetry, was greatly enhanced when programs such as Simplict90y (Mirada Medical, Denver, CO, USA) and SurePlan MRT (MIM Software Inc., Beachwood, OH, USA) became widely available.

Voxel-based dosimetry (VBD) is one of the more advanced dosimetric techniques being used. A major benefit is that VBD allows for the creation of dose volume histograms (DVH) and radiation dose maps. This in turn allows users to consider relative “hot” and “cold” spots within the tumor itself, rather than considering it as a homogenous compartment like in the partition method. This also gives clinicians the capability of dosing to the areas of tumor that receive the least amount of radiation. However, utilization and standardization have not necessarily followed these dosimetry advancements, with one study indicating that advanced dosimetry is only billed in about 1% of all US TARE interventions [8]. The various methods of dosimetry are summarized in Table 1.

This review explores the history of dosimetry and explains that, while numerous studies have shown a survival benefit for personalized dosimetry, there has been limited clinical adoption. Subsequently, it will identify challenges to adoption and assess future areas of investigation.

## 2. Methods

This narrative review followed a structured and transparent literature search strategy appropriate for non-systematic reviews. Comprehensive searches of PubMed, Embase, and Scopus were conducted using combinations of: ‘transarterial radioembolization’, ‘TARE’, ‘Y-90′, ‘yttrium-90′, ‘voxel-based dosimetry’, ‘partition dosimetry’, ‘personalized dosimetry’, ‘radiation segmentectomy’, and ‘dose–response’.

Searches were restricted to English-language human studies from 2000 to 2024. The included studies comprised clinical trials, prospective and retrospective cohorts, dosimetry analyses, and meta-analyses reporting absorbed dose metrics and clinical outcomes. Case reports, phantom-only studies, and cohorts with fewer than five patients were excluded unless uniquely informative. This review was conducted to synthesize and contextualize evolving dosimetric concepts across tumor types rather than to perform a formal systematic review. As such, the study selection emphasized methodological relevance, clinical impact, and contribution to the understanding of dose–response relationships in TARE. Several studies included in this review were authored by members of our group; these were selected according to the same inclusion criteria as all other studies and were incorporated based solely on their relevance and evidentiary value.

## 3. Dosimetry Methods

Dosimetry had modest beginnings when TARE first started being evaluated for efficacy in patients with primary or secondary malignancies of the liver [7,9,10]. Initially, with resin microspheres (SirSpheres, Sirtex, Woburn, MA, USA), the empirical method, which simply delivered 2.5 GBq of radiation regardless of liver or tumoral factors, was used as the paradigm in randomized trials. This method was quickly replaced, as patients developed unacceptable toxicities [11]. To replace it, semi-empirical methods, such as BSA and mBSA, were used and were demonstrated to be safe. However, this only provided a marginal improvement since tumor and non-tumor involving liver are not included in the calculations, and only the patient’s body surface area and percent tumor involvement of the liver, often a simple visual estimation by the provider, are used [11]. These semi-empiric methods are still employed by some today, despite recent prospective and retrospective studies indicating that utilization of these methods is linked to worse outcomes and decreased survival compared to other dosimetry methods [10,11,12,13]. This is likely due to not accounting accurately for the perfused and tumoral liver volume and the non-tumoral and tumoral absorbed radiation doses. Given these shortcomings, modern dosimetry practice has moved away from these methods, and the BSA/mBSA methodologies are no longer recommended [14].

Similarly, glass microspheres (Therasphere, Boston Scientific, Marlborough, MA, USA), use a different dosimetry technique, the single-compartment medical internal radiation dosimetry method often abbreviated as MIRD. This method, while accounting for actual perfused volume, unfortunately assumes the uniform distribution of particles throughout the perfused volume and reports the mean absorbed dose to the whole treated volume [2,15,16,17,18]. While many of the assumptions of the MIRD technique are not perfectly accurate, this method, which is the current on-label use of glass microspheres, is still superior to BSA given its more personalized calculation of the required activity [6,7,12].

Personalized or advanced dosimetry was originally referred to as the partition method, but now it also includes voxel-based dosimetry. Partition dosimetry, which is a multicompartment MIRD, differs from the single-compartment MIRD in that it recognizes the difference in dose distribution between the tumor and normal tissue compartments of the perfused volume [7]. The benefits on outcome measures from partition dosimetry have been highlighted in multiple studies [12,13]. However, it does still assume a uniform distribution of dose within each of the tumor and normal tissue compartments but does not adjust for intra-tumoral heterogeneity. While this is a conceptual step forward from either BSA/mBSA or single compartment MIRD, the partition method’s assumption of uniform distribution within the tumor compartment is inaccurate, which may impact the oncologic outcomes. This is commonly due to the tumor-to-normal ratio (TNR) often being visually or empirically estimated (e.g., always 4 for HCC) and not computed based on the Tc99m SPECT/CT obtained immediately after the planning/mapping angiogram. Voxel-based dosimetry differs from the partition method in that it recognizes that a dose does not distribute evenly in the tumor or normal tissue compartments and calculates dose at a voxel level. This granularity allows for the creation of dose volume histograms (DVHs). DVHs allow evaluation of the level of dose that reaches certain percentages of the tumor volume. For instance, one can calculate the “coldest” and “hottest” 5% tumor volumes (D95 or D5, respectively) and base their dose prescription accordingly. DVHs also allow users to evaluate what percentage of the tumor volume reaches a minimum dose threshold, such as 100 or 400 Gy (V100 or V400, respectively). Within the limitations of current radioembolization surrogates (i.e., macroaggregated albumin (MAA) and imaging protocols (SPECT/CT), this represents the most precise dosimetric method in use today. Unfortunately, it is associated with increased labor and time as well as the availability of dosimetry software, which is not ubiquitous. The importance of this dosimetric strategy was highlighted in a recent study, which demonstrated that, in HCCs treated with selective deliveries, the D95, or the 5% of the tumor receiving the least amount of radiation, was most predictive of complete pathologic necrosis (CPN) at explant [19]. CPN, in turn, has been tied to recurrence and outcome post-transplant and surgery, hence its importance as a surrogate measure of outcome [20].

Unfortunately, the data on tumor dose thresholds remain a main limitation of this technique. To overcome this, a randomized trial, Dosisphere-01, evaluated partition dosimetry, targeting specific dose targets to the tumor rather than single-compartment doses and demonstrated a clinically and statistically significant benefit to a more personalized approach for patients with locally advanced HCC [1]. This, combined with data that have indicated that significant T:N ratios exist even in more selective deliveries, has called into question the veracity of utilizing the MIRD dosimetry calculation method especially in large tumors and high tumor-to-normal liver volume ratios [21].

In general, while most will accept that voxel-based dosimetry provides the best information in terms of how the dose will be distributed, some have argued that advanced dosimetry is not essential to obtain excellent clinical results in certain clinical scenarios [2]. However, the evidence would support that advanced dosimetry is currently the standard for large HCCs [1], and voxel-based dosimetry may be established as the standard of care for other areas soon [4,19,20,21].

## 4. Current State of Dosimetry Based on Tumor Histology

### 4.1. Hepatocellular Carcinoma

HCC is perhaps the disease process in which the most supportive data for advanced dosimetry exist. For very large HCCs, the landmark study DOSISPHERE-01 was published in 2021 [1]. This study was a phase 2 randomized controlled trial, which aimed to evaluate treatment with single compartmental MIRD at 120 Gy to the perfused volume compared to multicompartmental MIRD (partition) with a target of more than 205 Gy, ideally at least 250 Gy, to the tumor. The study utilized Simplicit90y (Mirada Medical, Denver, CO, USA) for the partition method arm and was designed to enroll 254 patients. However, during the first planned interim analysis after 60 patients had been enrolled, the study was deemed no longer ethical to continue, as the difference in the overall survival (OS) was so profound. In the intention to treat (ITT) analysis the personalized dosimetry arm demonstrated an OS of 26.6 months (95% CI: 11.7–NR) versus 10.7 months (95% CI: 6–16.8) in the standard arm (HR = 0.421 [95% CI: 0.215–0.826]; *p* = 0.0096). The more recent publication of the 5-year follow-up data also demonstrated maintenance of this OS benefit with a median OS of 24.8 months (95% CI, 11–36.5) in the group receiving advanced dosimetry and 10.7 months (95% CI, 6–14.9) in the standard dosimetry group (HR, 0.51; 95% CI, 0.29–0.9; *p* = 0.020) [22]. With multivariate analysis in this population with large tumors and a high percentage of portal vein tumor thrombus, downstaging to secondary resection was specifically implicated for prolonged overall survival. Importantly, the increased treatment response did not result in increased adverse events. This landmark study has made advanced or personalized dosimetry the standard of care for this patient population. The TARGET study similarly evaluated larger tumors with almost 50% being at least 8 cm. This study demonstrated median OS was 36.7 months when the tumor absorbed dose was at least 300 Gy. This is in comparison to a median OS of 16.1 months when the tumor absorbed dose was less than 200 Gy [23].

When turning to selective deliveries, or segmentectomies as they are often referred to, current practice is often driven by the LEGACY data [2]. In a subset of LEGACY patients, Gabr et al. evaluated 45 patients with HCCs <8 cm treated with selective Y90 delivery and found that all patients who had received >400 Gy to the segment or perfused volume had CPN after explant [24]. Similarly, Toskich et al. performed a single center retrospective analysis of HCC patients who underwent radiation segmentectomy prior to liver transplantation for 33 patients. He found that CPN was present in 53% and 75% of tumors that underwent >190 Gy and >500 Gy radiation segmentectomy using MIRD dosimetry [25]. In another study along the same lines, Montazeri et al. evaluated patients who underwent radiation segmentectomy and found that those who had treatment intensification had higher rates of CPN (76%) compared to the baseline cohort (49%) [26]. While several reasons for this discrepancy in CPN at high doses might exist, the presence of significant TNR differences do exist even with selective deliveries, in addition to the heterogeneity of dose within the tumor compartment [20,21]. In fact, a recent study of explanted livers in a similar patient cohort to both LEGACY and the follow-up studies found that a D95 >719 Gy was most predictive of achieving CPN [19]. These data suggest that utilization of voxel-based dosimetry could be of use even in the selective delivery setting when treating HCC. Furthermore, these data also suggest that the blanket approach of delivering a mean absorbed dose of 400 Gy to every perfused volume for selective treatments may not be adequate, as the tumor volume-to-perfused/angiosome volume ratio will ultimately alter the tumor dose.

### 4.2. Intrahepatic Cholangiocarcinoma

Several studies have supported the efficacy of TARE in the setting of intrahepatic cholangiocarcinoma (iCCA) [4,5,27,28,29,30,31,32,33]. Unfortunately, the dosimetry data are less robust and more variable compared to HCC, but emerging evidence highlights its importance. Evidence from a phase 2 clinical trial by Edeline et al. demonstrated that TARE combined with chemotherapy as a first-line treatment for locally advanced iCCA resulted in a median OS of 22 months and a median PFS of 14 months using MIRD dosimetry with the option of treatment personalization to optimize outcomes [5]. The median dose to the tumor was 317 Gy, which resulted in a best response rate of 41% by RECIST1.1 and 93% by the Choi criteria. Additionally, nine patients were downstaged to surgery. Comparatively, Jia et al. evaluated 24 patients with unresectable iCCA who underwent resin Y90 using the BSA dosimetry after failure of first line chemotherapy. The authors noted partial response, stable disease, and progressive disease in 36.4%, 45.5%, and 18.2% of patients by mRECIST [27].

Another study by Manceau et al. evaluated 35 patients with iCCA who underwent glass Y90 with partition dosimetry in combination with chemotherapy. With a mean tumor dose of 310 Gy, the response rate using EASL was 96%. A median progression free survival of 12.7 months and median overall survival of 28.6 months were noted in this cohort. The mean healthy liver dose was under 80 Gy and was not associated with permanent liver toxicity [28]. Young et al. retrospectively evaluated 26 iCCA tumors treated in 20 patients and found that a threshold of >541.7 Gy to the tumor with glass Y90 was most predictive of a complete radiologic response (CR) [4]. This study also confirmed that patients achieving CR had improved overall survival (OS), underscoring the role of dosimetry in optimizing outcomes. In a separate study, Edeline et al. compared existing prospective studies utilizing chemotherapy alone vs. combination radioembolization plus chemotherapy. They found a statistically significant OS and PFS benefit for the combination therapy group, further supporting the use of Y90 in combination with chemotherapy in iCCA [34].

Along similar lines supporting the use of personalized dosimetry in iCCA, a multicenter study by Levillain et al. evaluated personalized radioembolization in 58 patients with unresectable and chemorefractory iCCA with resin microspheres. BSA and more personalized partition models were used for dosimetry. They noted a median OS of 10.3 months in the entire cohort, but more importantly they noted a significantly shorter OS in patients who were treated with BSA compared to the partition model (5.5 vs. 14.9 months, HR = 2.52, 95% CI: 1.23–5.16). They also noted a higher mean radiation dose to the tumors in the partition model compared to the BSA model, 86 Gy vs. 38 Gy, respectively [29]. Similarly, Reimer et al. showed an OS and PFS benefit for first-line TARE plus systemic therapy over TARE alone as well as a benefit for partition dosimetry over BSA dosimetry [13].

In the context of early stage iCCA, a recent retrospective study by Gupta et al. validated the safety and effectiveness of radiation segmentectomy (RS) and modified radiation lobectomy (mRL) in 15 patients with unresectable solitary iCCA without extrahepatic disease or vascular involvement. The median treatment dose was 308.2 Gy (range, 194.2–879.3 Gy), with four patients receiving doses >400 Gy. The study reported a 3-month best objective response rate of 60% by RECIST 1.1 criteria, with three patients who went on to have surgery achieving complete pathologic necrosis. The median OS was 72 months, with 1, 3, and 5-year OS rates of 100%, 73.3%, and 50.3%, respectively. These outcomes suggest that RS and mRL, guided by advanced dosimetry, may offer a viable therapeutic option for early-stage iCCA, comparable to surgical resection [30].

With regard to iCCA, there is much variability in the dosimetry methods used, doses given, and outcomes. Multiple studies have been summarized above, most with positive outcomes with higher radiation doses and with the use of personalized dosimetry. Bourien et al. further supported this, finding that a dose >260 Gy to the tumor resulted in a significantly higher OS (28.2 vs. 11.4 months). They utilized glass microspheres with MIRD dosimetry targeting 80–150 Gy to the targeted liver volumes [35].

While the data for iCCA are less extensive than for HCC, these studies collectively argue for the critical role of personalized dosimetry in improving radiologic and pathologic responses, as well as survival outcomes, in iCCA patients. Further studies are needed to standardize the dose thresholds and expand the evidence base, particularly for selective deliveries and early-stage disease.

### 4.3. Metastatic Colorectal Cancer (mCRC)

Metastatic colorectal carcinoma (mCRC), comparatively, has been evaluated in the literature more extensively than other metastases, but less than primary liver malignancies. A recent RCT looking at patients treated with standard of care chemotherapy plus or minus TARE demonstrated longer hepatic and overall progression free survival as well as objective response rates in the TARE cohort [3]. These data established the role of TARE in properly selected patients with mCRC [3]. In perhaps the strongest dosimetry-centered data, the TACOME trial in 176 patients demonstrated an estimated tumor absorbed dose difference between responders and nonresponders at 200 Gy and 104 Gy, respectively, *p* = 0.001. There was no difference in treatment response with relation to KRAS mutation or location of primary tumor, but the median microsphere concentration in the tumor volume also demonstrated statistical significance. An estimated tumor absorbed dose of 152 Gy also met statistical significance with respect to the median OS, with 18.1 months for responders versus 12.8 months for non-responders [36].

Comparatively, Alsultan et al. evaluated the metabolic tumor response with pre and post glass Y90 treatment positron emission tomography (PET)/CT to determine the tumor response [37]. MIRD dosimetry was used for the treatment, but post treatment dosimetry was performed on PET/CT. This retrospective analysis of 31 patients and 85 mCRC lesions found that a minimum tumor absorbed dose of 139 Gy predicted the 3-month metabolic response most accurately (89% specificity and 77% sensitivity). Additionally, a mean tumor absorbed dose of 196 Gy and 177 Gy was seen in patients that had a complete response and a partial response, respectively. These doses were, importantly, 94% and 74% higher than patients that had progressive disease.

Unfortunately, while few data are available in the setting of selective deliveries for mCRC, there are studies that evaluate the effect of dosimetry in colorectal metastases using both voxel-based dosimetry and other dosimetric predictors of outcomes [38,39,40]. One study by Dimopoulos et al. evaluated the dosimetric response of mCRC to yttrium-90 radiation segmentectomy in 36 heavily pretreated patients with 57 tumors. The study found that a mean tumor dose (MTD) ≥400 Gy and a 5 mm margin mean absorbed dose (MMAD) ≥350 Gy were independent predictors of prolonged local tumor progression-free survival (LTPFS), with a one-year LTPFS rate of 83.3% for tumors meeting these thresholds (*p* = 0.006). Additionally, no instances of local tumor progression were observed when at least 95% of the tumor volume received ≥300 Gy. Complete radiographic and metabolic responses, assessed by RECIST 1.1 and PERCIST criteria, were significantly associated with prolonged LTPFS (*p* < 0.001 and *p* = 0.038, respectively). These findings underscore the critical role of voxel-based dosimetry in optimizing RS outcomes for CLM, emphasizing the importance of achieving high tumor and margin doses to enhance local tumor control [39].

Another study by Dimopoulos et al. analyzed 231 patients with mCRC undergoing 329 Y90 radioembolization (glass and resin) sessions, focusing on dosimetric predictors of outcomes. The study found that a weighted tumor absorbed dose covering at least 90% of the tumor (WTD90) ≥120 Gy was an independent predictor of improved overall survival (OS) (*p* = 0.043). Additionally, covering at least 50% of the tumor positively impacted target liver progression-free survival (TLPFS) (*p* = 0.003). Response assessments using RECIST 1.1 and PERCIST criteria showed that higher tumor doses correlated with improved local tumor control and survival, again reinforcing the critical role of personalized dosimetry in optimizing TARE outcomes for CLM [40].

### 4.4. Metastatic Neuroendocrine Tumor and Breast Cancer

While the dosimetry data for metastatic neuroendocrine tumors (NET) and breast cancer are less extensive than for HCC, mCRC, or iCCA, emerging studies underscore the importance of personalized dosimetry in optimizing TARE outcomes for these malignancies [41,42,43]. For NET, TARE has been recognized as an effective treatment for liver metastases, particularly in patients with unresectable disease. A retrospective study by Ebbers et al. analyzed 26 patients with 128 NET liver metastases treated with Y-90 glass microspheres. Dosimetry evaluation was performed on post radioembolization PET/CT. A statistically significant difference was found with a median dose for responding tumors of 194 Gy and for nonresponding tumors of 114 Gy, *p* < 0.001. Using a cutoff of 150 Gy, the median OS differed significantly as well, at 29.9 months versus 12 months [41].

Similarly, a study by Chansanti et al. evaluated 55 tumors in 15 patients with NET liver metastases treated with Y-90 resin microspheres, using SPECT/CT partition dosimetry. In the per patient analysis, at initial follow-up, there was a 47% ORR by mRECIST. The mean estimated tumor absorbed dose was significantly higher in responders, 207 Gy versus 114 Gy, *p* = 0.049. In the per tumor analysis, the ORR was 65.4%, and a much higher mean estimated tumor dose in responders compared to non-responders was again seen (286 Gy vs. 128 Gy, *p* = 0.0004). A threshold dose of 191.3 Gy corresponding to 83% sensitivity and 93% specificity for response was identified. The tumor burden and grade were not associated with the response.

Watanabe et al. evaluated 43 patients who underwent glass Y90 radioembolization for NET metastases to the liver. Treatment doses were determined using MIRD dosimetry. Post hoc analysis using multicompartment (partition) dosimetry using Simplicit90Y software (Mirada Medical) was performed to identify the mean absorbed dose (MAD). The median values for responder vs. nonresponder lesions were 237.6 Gy and 120.2 Gy. Interestingly, during their univariate analysis for hepatic PFS, they found that tumor grade and origin were significant prognostic factors, but MAD was not. However, in the univariate and multivariate analysis for global PFS they found that MAD was a significant prognosticator [43]. A recent retrospective study by Gordon et al. investigated the efficacy of yttrium-90 glass radiation segmentectomy in 18 patients with NET liver metastases. Primary sites included bowel, pancreatic, and pulmonary, as well as grades 1–3 disease. A median dose of 235.3 Gy to a median treatment volume of 163.3 mL was delivered. Objective response rates of 83% by RECIST 1.1 criteria and 100% by mRECIST criteria were reported, with a complete response observed in four patients, a partial response in 11, and stable disease in three by RECIST 1.1. These findings continue to highlight the potential of dosimetry-guided radiation segmentectomy to achieve high response rates in NET liver metastases, supporting the use of personalized dosimetry to optimize therapeutic outcomes, particularly in selective delivery settings [44].

For breast cancer liver metastases, the data are more limited but still support the role of dosimetry-guided TARE. A retrospective study by Ridouani et al. evaluated 60 patients with pre and post glass or resin TARE PET/CT. Of the 60 patients, 46 had objective response with responders receiving a median of 167 Gy tumoral dose compared to nonresponders who received 54 Gy (*p* <0.001). Treatment in this study was performed using MIRD dosimetry for glass and body surface area with tumor volume for resin, while the post treatment absorbed dose estimation was performed on SPECT/CT [45].

While the data for NET and breast cancer are less robust than for HCC or iCCA, these studies collectively highlight the critical role of advanced dosimetry in achieving optimal therapeutic outcomes. However, further research is needed to establish standardized dose thresholds and to validate these findings in larger prospective studies, particularly for selective deliveries and patients with varying tumor burdens.

Table 2 summarizes the various larger studies by tumor histology.

## 5. Evolving Concepts in Dosimetry

While much of the historical discussion and evidence around advanced dosimetry has been centered around recognizing the differential dose between the tumor and normal tissue compartments of the perfused volume, further nuance is being evaluated with voxel-based dosimetry. One concept, which was alluded to above and is beginning to take shape, consists of dosing to the “coldest” portions of the tumor or the portions that receive the least radiation. For instance, a recent retrospective single center study by Pianka et al. included 41 patients who were treated for HCC with selective TARE delivery. The authors demonstrated that a D95 >719 Gy was most predictive of CPN on explant [19]. As discussed above, this perhaps explains why discrepancies occurred between prior pathologic analyses. Prior studies did not control for differential perfusion between normal tissue and tumor nor the distribution of the dose within the tumor itself [2,20,25,26]. Another recent study looked at radiologic responses and found that D60, D70, D80, D90, and D99 are predictive of radiologic response [46]. These data then begin to support the concept of differential blood flow to various portions of the tumor and the variation which can be seen between different tumors. Furthermore, dosing to the “coldest” portions of the tumor or ensuring that all or much of the tumor reaches a certain dose threshold may provide the pathway towards optimizing outcomes. For this reason, the authors of this manuscript conjecture that dosing to the “coldest” 5–20%, or D95-D80, or ensuring that the entirety of the tumor receives at least 400 Gy (V400) will likely be the future of dosimetry. The appeal of this method is enhanced when considering that TARE is the only radiation treatment in which utilization of post treatment imaging can document dose distribution and diagnose undertreatment or predict outcome. This contrasts with other radiation treatments such as stereotactic body radiation therapy (SBRT) where internal motion, particularly in the abdomen, does not allow for documentation of where the dose was ultimately delivered.

Studies have highlighted that CR on imaging following Y90 radiation segmentectomy does not always correlate with complete pathologic necrosis (CPN) in treated tumors. The LEGACY study reported that all patients with HCC receiving >400 Gy to the segment achieved CPN, yet a follow-up study with a similar cohort found only 75% achieved CPN despite CR on imaging, suggesting discrepancies due to heterogeneous dose distribution within the tumor [2,25]. These findings underscore that imaging-based CR may overestimate the pathologic response due to limitations in detecting microscopic residual disease, which emphasizes the importance of voxel-based dosimetry to ensure adequate tumor dose coverage to predict and diagnose both imaging and pathologic response.

Microsphere density and microsphere activity have also emerged as important considerations [19,25,26,47]. Microsphere density is typically utilized to describe how many microspheres per milliliter (mL) of tissue are delivered, while microsphere activity typically refers to the radiation activity that each sphere carries. These two concepts are intimately linked in an inverse relationship. For example, if the prescribed activity delivered to a perfused volume is kept steady, increasing the microsphere density will decrease the activity per microsphere and vice versa. These two variables have garnered more attention over the last several years, with multiple studies on pathologic response after explant in HCC patients suggesting that specific activity was most predictive of achieving CPN [25,26]. Another recent study evaluating the variability of the tumor absorbed dose in HCC patients suggested that the microsphere density had a significant impact on TNR [21]. Several studies have tried to evaluate the ideal microsphere load, resulting in the development of an important concept: larger microsphere loads will simply result in larger clusters of microspheres in existing locations of the tumor as opposed to adding significant clusters of particles to new locations within the tumor [19,44,46,47,48]. Additionally, each intratumoral location in which microspheres will cluster has a relative number of microspheres it can accept. These locations eventually become saturated, leading to increased microspheres flowing towards the normal parenchyma. This in turn explains why some data have suggested that higher microsphere density tends to drive down the TNR. While this concept helps us understand how perfused volumes will react as the microsphere density is increased, it is still difficult to identify an “ideal” microsphere load at this time. However, it is possible to say that it is likely beneficial to have at least 5000 microspheres/mL of tumoral tissue [49,50]. It should also be noted that this density can be achieved using any of the commercially available products while prescribing a dose in a manner consistent with the literature [46].

## 6. Adoption Barriers and Solutions

While the current literature continues to generate evidence to support the use of voxel-based or advanced dosimetry, adoption has lagged based on claims data [8]. A recent survey of CMS data suggests that only 1% of all TARE interventions in the US result in a CPT code submission for advanced dosimetry techniques. While it is likely that some centers are performing these dosimetry measurements and not performing the required documentation nor submitting the claims, it is also likely that the vast majority of cases are not being treated with advanced dosimetry. There are several logistical reasons why adoption has not occurred. The first potential reason is that new evidence often takes time to change practice, either because providers are unaware or are skeptical of the data. Even after accepting the necessity for advanced dosimetry to drive outcomes, though, further resources are needed for this capability such as software platforms, expertise, and time. While there are several available programs on the market including Simplict90y (Mirada Medical, Denver, CO, USA) and SurePlan MRT (MIM Software Inc, Beachwood, OH, USA), obtaining and implementing them in the heavily guarded health care environment can represent a barrier. Furthermore, both programs have associated learning curves, and these can be prolonged in practices in which TARE is performed infrequently. Finally, physicians have increasingly diminishing time, and time spent on dosimetry is often at the expense of other patient care and revenue generating activities despite reimbursement for such work being economically favorable in the U.S. These limitations are intensified by the lack of access to and shortages of interventional radiologists [51]. These disparities are also present internationally as utilization of non BSA dosimetry has been demonstrated in 13% to 50% in US and European publications, respectively [13,52].

While these issues are daunting, several different solutions are available particularly in the US. There are now “dosimetry as a service” companies being created. These companies offer the ability to outsource the labor and workflow involved in dosimetry. Pre and post TARE dosimetry including segmentation, lung shunt calculation, and TNR determination as well as DVH creation, standardization, and quality control can all be performed. They can also provide evidence-based protocols to provide suggested dosing strategies, alleviating some of the burden for physicians who struggle to find time to keep up with the literature. Some larger systems have also looked at creating a dosimetry core, where trained technologists provide much of the same work as offered by a commercial company. Alternatively, other systems have designated a single provider to perform all the dosimetry work for the group. Nevertheless, the evidence has clearly indicated that advanced dosimetry provides better outcomes for patients, and recognition of this by payers has increased incentives in the US.

## 7. Limitations of Existing Evidence and Dosimetric Approaches

### 7.1. Study Design Limitations

Much of the evidence supporting advanced dosimetry in TARE is derived from retrospective analyses and single center experiences, frequently from high volume institutions. Some prospective studies and randomized controlled trials exist but are fewer in number and often smaller in data size. This introduces center-level selection and publication bias, which can limit the generalizability.

### 7.2. Imaging and Dosimetry Variability

There are several limitations within the current body of evidence supporting advanced dosimetry. Significant heterogeneity exists across institutions regarding imaging acquisition, reconstruction algorithms, segmentation protocols, dose-kernel modeling, and utilization of DVH parameters such as D90 vs. D95 and V100 vs. V400, This complicates cross-study comparison and can limit standardization.

### 7.3. Mismatch Between MAA and Y90 Distribution

The reliance on technetium-99 m macroaggregated albumin as a surrogate for microsphere distribution remains an inherent limitation of current dosimetric planning. This remains a persistent challenge, particularly in less selective treatments where physiologic or catheter-position differences also may distort tumor-to-normal uptake estimates [53].

### 7.4. Generalizability and Access Barriers

Advanced dosimetry requires specialized software, technical expertise, and additional time investment, which may limit adoption in lower volume or resource constrained settings. Voxel-based dosimetry also remains sensitive to platform-dependent variability; commercial software packages (e.g., MIM, Mirada) use proprietary reconstruction kernels and segmentation tools that are not directly interchangeable. These barriers may contribute to disparities in access and may slow widespread implementation despite rapidly growing evidence of clinical benefit. These limitations emphasize the need for standardized reporting frameworks, harmonized imaging and treatment protocols, and multicenter prospective studies.

## 8. Conclusions

Accumulating evidence across multiple hepatic malignancies supports the clinical value and importance of personalized dosimetry in transarterial radioembolization. First, individualized dose planning with advanced dosimetry is associated with improved tumor response and survival in selected patients compared with empiric, semi-empiric, or single-compartment dose–based approaches. Second, voxel-based dosimetric metrics offer superior characterization of intratumoral dose heterogeneity and may better predict pathologic and radiologic outcomes than mean absorbed dose alone. Third, broader adoption of advanced dosimetry will require continued efforts toward standardization, workflow efficiency, and scalable implementation.

As dosimetric techniques continue to evolve, prospective validation and harmonized reporting will be essential to translate these advances into consistent improvements in patient outcomes.

## Figures and Tables

**Table 1:** Comparison of dosimetry methods for transarterial radioembolization.

Dosimetry Method	Key Concepts	Advantages	Limitations
BSA/Modified BSA (mBSA)	Activity derived from body surface area and estimated % tumor involvement	Historical use with resin microspheres, ease of use	Does not incorporate perfused or tumor volume
Single-Compartment MIRD	Computes mean absorbed dose to the whole perfused liver volume	Historical use with glass microspheres, ease of use	Does not differentiate tumor vs. normal liver dose
Partition Dosimetry (Multicompartment MIRD)	Separates tumor, normal liver, and lung compartments based on uptake ratios	Accounts for tumor and normal liver separately, supports dose targeting	Unable to assess heterogeneity within tumor, relies on accurate tumor to normal ratio (TNR) estimation
Voxel-Based Dosimetry (VBD)	Calculates absorbed dose per voxel and generates DVHs (D95, V100, V400)	Highest precision, identifies hot/cold regions, predicts pathologic and radiologic response	Time-intensive, requires specialized software, dependent on high-quality imaging

Legend Key: mBSA—modified body surface area, MIRD—medical internal radiation dose, DVH—Dose volume histograms, VBD—voxel based dosimetry.

**Table 2:** Tumor-specific dose thresholds for transarterial radioembolization.

Study	Design	N	Microsphere	Response Criteria	Key Outcome
**Hepatocellular Carcinoma**					
Garin et al. (DOSISPHERE-01) [1]	RCT MIRD (120 Gy) vs. Partition (205 Gy)	60	Glass; Lobar	RECIST 1.1	OS 26.6 (partition) vs. 10.7 (MIRD) months No increased AEs
Salem et al. (LEGACY) [2]	Retrospective MIRD	162	Glass; Selective	mRECIST	Localized mRECIST 88% ORR with 76% demonstrating response ≥ 6 months
Pianka et al. [19]	Retrospective Voxel-based Explant Pathology	41	Glass; selective	Pathologic necrosis	D95 predicts CPN D95: 813 Gy (CPN) vs. 232 Gy (w/o CPN)
**Intrahepatic Cholangiocarcinoma**					
Edeline et al. (MISPHEC) [5]	Prospective single arm MIRD 120 Gy lobar, personalized dosimetry authorized	41	Glass; Lobar	RECIST 1.1	OS 22 months 317 Gy median tumor dose Choi response rate 93%
Young et al. [4]	Retrospective BSA, MIRD, voxel-based	20	Glass/Resin	EASL	CR associated 542 Gy tumor dose (glass, post Y90 dosimetry)
Levillain et al. [29]	Retrospective BSA and partition	58	Resin	PERCIST	Median OS Partition, 14.9 months BSA, 5.5 months
**Metastatic Colorectal Cancer**					
Soydal et al. (TACOME) [36]	Retrospective Single-compartment or multicompartment dosimetry	176	Glass	PERCIST	Responders: 200 Gy Nonresponders: 104 Gy
Alsultan et al. [37]	Retrospective MIRD	31	Glass	PERCIST	CR 196 Gy PR 177 Gy (post Y90 dosimetry)
Dimopoulos et al. [39]	Retrospective MIRD	36	Glass	RECIST/PERCIST	≥400 Gy—predictor of LTPFS (post Y90 dosimetry)
**Neuroendocrine (NET) and Metastatic Breast Cancer**					
Ebbers et al. (NET) [41]	Retrospective MIRD	26	Glass	RECIST1.1	Absorbed dose (Gy) (post Y90 dosimetry): - Responder: 170 - Stable: 101 - Progressive: 67
Chansanti et al. (NET) [42]	Retrospective Partition	15	Resin	mRECIST	Absorbed dose (Gy): - Responder: 286 - Nonresponder: 128
Ridouani et al. [45]	Retrospective BSA and MIRD	64	Glass/Resin	Modified PERCIST	Absorbed dose (Gy) (post Y90 dosimetry): - Responder: 167 - Nonresponder: 54

Legend Key: RCT—randomized controlled trial, MIRD—medical internal radiation dose, BSA—body surface area, RECIST—response evaluation criteria in solid tumors, mRECIST—modified response evaluation criteria in solid tumors, CPN—complete pathologic necrosis, EASL—European Association for Study of Liver, PERCIST—PET response criteria in solid tumors.

## Data Availability

Not applicable.

## Abbreviations

The following abbreviations are used in this manuscript:

HCC	Hepatocellular Carcinoma
TARE	Transarterial Radioembolization
iCCA	Intrahepatic Cholangiocarcinoma
mCRC	Metastatic Colorectal Cancer
NET	Neuroendocrine Tumor
D95	Minimum Dose Received by 95% of the Target Volume/Tumor
DVH	Dose Volume Histograms
BSA	Body Surface Area
mBSA	Modified Body Surface Area
CLM	Colorectal Liver Metastases
CMS	Center for Medicare & Medicaid Services
CPT	Current Procedural Terminology
CPN	Complete Pathologic Necrosis
CR	Complete (Radiologic) Response
CT	Computed Tomography
HR	Hazard Ratio
ITILD50	Intention to Irradiate Liver Dose Covering at Least 50% of the Tumor
ITT	Intention to Treat
MIRD	Medical Internal Radiation Dose
MMAD	Margin Mean Absorbed Dose
MTD	Mean Tumor Dose
OS	Overall Survival
PERCIST	PET Response Criteria in Solid Tumors
PFS	Progression Free Survival
RECIST 1.1	Response Evaluation Criteria in Solid Tumors, version 1.1
RS	Radiation Segmentectomy
SBRT	Stereotactic Body Radiation Therapy
SHR	Subdistribution Hazard Ratio
T:N (TNR)	Tumor-to-Normal Ratio
TLPFS	Target Liver Progression Free Survival
WTD90	Weighted Tumor Absorbed Dose Covering at Least 90% of Tumor
mRL	Modified Radiation Lobectomy

## References

[1] Garin E., Tselikas L., Guiu B., Chalaye J., Edeline J., de Baere T., Assénat E., Tacher V., Robert C., Terroir-Cassou-Mounat M. (2021). Personalized versus standard dosimetry approach of selective internal radiation therapy in patients with locally advanced hepatocellular carcinoma (DOSISPHERE-01): A randomised, multicentre, open-label phase 2 trial. Lancet Gastroenterol. Hepatol..

[2] Salem R., Johnson G.E., Kim E., Riaz A., Bishay V., Boucher E., Fowers K., Lewandowski R., Padia S.A. (2021). Yttrium-90 radioembolization for the treatment of solitary, unresectable HCC: The LEGACY study. Hepatology.

[3] Mulcachy M.F., Mhvash A., Pracht M., Montazeri A.H., Bandula S., Martin R.C. (2021). Radioembolization with Chemotherapy for Colorectal Liver Metastases: A Randomized, Open-Label, International, Multicenter, Phase III Trial. J. Clin. Oncol..

[4] Young S., Torkian P., Flanagan S., D’sOuza D., Sanghvi T., Golzarian J. (2023). Intrahepatic cholangiocarcinoma: A dose threshold evaluation in those undergoing transarterial radioembolization. J. Gastrointest. Oncol..

[5] Edeline J., Touchefeu Y., Guiu B., Farge O., Tougeron D., Baumgaertner I., Garin E. (2020). Radioembolization Plus Chemotherapy for First-line Treatment of Locally Advanced Intrahepatic Cholangiocarcinoma: A Phase 2 Clinical Trial. JAMA Oncol..

[6] Villalobos A., Pisanie J.L.D., Gandhi R.T., Kokabi N. (2024). Yttrium-90 Radioembolization Dosimetry: Dose Considerations, Optimization, and Tips. Semin. Interv. Radiol..

[7] Young S., Goldberg D., Hannallah J., Stuycken L., Woodhead G. (2024). Advancing Radioembolization Through Personalized Dosimetry. Adv. Clin Rad..

[8] CMS CMS Standard Analytical Files. https://www.cms.gov/data-research/files-for-order/limited-data-set-lds-files/standard-analytical-files-medicare-claims-lds.

[9] Van Hazel G., Blackwell A., Anderson J., Price D., Moroz P., Bower G., Cardaci G., Gray B. (2004). Randomised phase 2 trial of SIR-Spheres plus fluorouracil/leucovorin chemotherapy versus fluorouracil/leucovorin chemotherapy alone in advanced colorectal cancer. J. Surg. Oncol..

[10] Kao Y.H., Lichtenstein M. (2016). Origin, dosimetric effect and clinical limitations of the semi-empirical body surface area method for radioembolization using yttrim-90 resin microspheres. J. Med. Imagin. Radiat. Oncol..

[11] Webster L.A., Villalobos A., Majdalany B.S., Bercu Z.L., Gandhi R.T., Kokabi N. (2021). Standard Radiation Dosimetry Models: What Interventional Radiologists Need to Know. Semin. Interv. Radiol..

[12] Schaefer N., Grozinger G., Pech M., Pfammatter T., Soydal C., Arnold D., Kolligs F., Maleux G., Munneke G., Peynircioglu B. (2022). Prognostic Factors for Effectiveness Outcomes After Transarterial Radioembolization in Metastatic Colorectal Cancer: Results From the Multicentre Observational Study CIRT. Clin. Colorectal Cancer.

[13] Reimer P., Vilgrain V., Arnold D., Balli T., Golfieri R., Loffroy R., Mosconi C., Ronot M., Sengel C., Schaefer N. (2024). Factors Impacting Survival After Transarterial Radioembolization in Patients with Unresectable Intrahepatic Cholangiocarcinoma: A Combined Analysis of the Prospective CIRT Studies. Cardiovasc. Interv. Radiol..

[14] Levillain H., Bagni O., Deroose C.M., Dieudonné A., Gnesin S., Grosser O.S., Kappadath S.C., Kennedy A., Kokabi N., Liu D.M. (2021). International recommendations for personalised selective internal radiation therapy of primary and metastatic liver diseases with yttrium-90 resin microspheres. Eur. J. Nucl. Med. Mol. Imaging.

[15] Knight G.M., Gordon A.C., Gates V., Talwar A., Riaz A., Salem R., Lewandowski R. (2023). Evolution of Personalized Dosimetry for Radioembolization of Hepatocellular Carcinoma. J. Vasc. Interv. Radiol..

[16] Gulec S.A., McGoron A.J. (2022). Radiomicrosphere Dosimetry: Principles and Current State of the Art. Semin. Nucl. Med..

[17] Villalobos A., Soliman M.M., Majdalany B.S., Schuster D.M., Galt J., Bercu Z.L., Kokabi N. (2020). Yttrium-90 Radioembolization Dosimetry: What Trainees Need to Know. Semin. Interv. Radiol..

[18] Keane G., Lam M., Jong H.D. (2021). Beyond the MAA-Y90 Paradigm: The Evolution of Radioembolization Dosimetry Approaches and Scout Particles. Semin. Interv. Radiol..

[19] Pianka K.T., Barahman M., Minocha J., Minocha J., Redmond J.W., Schnickel G.T., Rose S.C., Fowler K.J., Berman Z.T. (2024). Voxel-based tumor dose correlates to complete pathologic necrosis after transarterial radioembolization for hepatocellular carcinoma. Eur. J. Nucl. Med. Mol. Imaging.

[20] Nie R., Chen F., Provencio M., Wang Y., Ende T.v.D., van Laarhoven H.W., Yuan S., Pless M., Hayoz S., Zhou Z. (2023). Predictive value of radiological response, pathological response and relapse-free survival for overall survival in neoadjuvant immunotherapy trials: Pooled analysis of 29 clinical trials. Eur. J. Cancer.

[21] Young S., Chen T., Flanagan S., Golzarian J., Sanghvi T. (2022). Realized tumor to normal ratios in hepatocellular carcinoma patients undergoing transarterial radioembolization: A retrospective evaluation. Eur. Radiol..

[22] Garin E., Tselikas L., Guiu B., Chalaye J., Rolland Y., de Baere T., Assenat E., Tacher V., Palard X., Déandreis D. (2020). Long-Term Overall Survival After Selective Internal Radiation Therapy for Locally Advanced Hepatocellular Carcinomas: Updated Analysis of DOSISPHERE-01 Trial. J. Nucl. Med..

[23] Lam M., Garin E., Maccauro M., Kappadath S.C., Sze D.Y., Turkmen C., Cantasdemir M., Haste P., Herrmann K., Alsuhaibani H.S. (2022). A global evaluation of advanced dosimetry in transarterial radioembolization of hepatocellular carcinoma with Yttrium-90: The TARGET study. Eur. J. Nucl. Med. Mol. Imaging.

[24] Gabr A., Riaz A., Johnson G.E., Kim E., Padia S., Lewandowski R.J., Salem R. (2021). Correlation of Y90-absorbed radiation dose to pathological necrosis in hepatocellular carcinoma: Confirmatory multicenter analysis in 45 explants. Eur. J. Nucl. Med. Mol. Imaging.

[25] Toskich B., Vidal L.L., Olson M.T., Lewis J.T., LeGout J.D., Sella D.M., Patel T.C. (2021). Pathologic Response of Hepatocellular Carcinoma Treated with Yttrium-90 Glass Microsphere Radiation Segmentectomy Prior to Liver Transplantation: A Validation Study. J. Vasc. Interv. Radiol..

[26] Montazeri S.A., De la Garza-Ramos C., Lewis A.R., Lewis J.T., LeGout J.D., Sella D.M., Paz-Fumagalli R., Devcic Z., Ritchie C.A., Frey G.T. (2022). Hepatocellular carcinoma radiation segmentectomy treatment intensification prior to liver transplantation increases rates of complete pathologic necrosis: An explant analysis of 75 tumors. Eur. J. Nucl. Med. Mol. Imaging.

[27] Jia Z., Paz-Fumagalli R., Frey G., Sella D.M., McKinney J.M., Wang W. (2017). Resin-based Yttrium-90 microspheres for unresectable and failed first-line chemotherapy intrahepatic cholangiocarcinoma: Preliminary results. J. Cancer Res. Clin. Oncol..

[28] Manceau V., Palard X., Rolland Y., Pracht M., Le Sourd S., Laffont S., Boudjema K., Lievre A., Mesbah H., Haumont L.-A. (2018). A MAA-based dosimetric study in patients with intrahepatic cholangiocarcinoma treated with a combination of chemotherapy and 90Y-loaded glass microsphere selective internal radiation therapy. Eur. J. Nucl. Med. Mol. Imaging.

[29] Levillain H., Derijckere I.D., Ameye L., Guiot T., Braat A., Meyer C., Vanderlinden B., Reynaert N., Hendlisz A., Lam M. (2019). Personalised radioembolization improves outcomes in refractory intra-hepatic cholangiocarcinoma: A multicenter study. Eur. J. Nucl. Med. Mol. Imaging.

[30] Gupta A.N., Serhal M., Gordon A.C., Gabr A., Kalyan A., Kulik L., Sato K.T., Riaz A., Hohlastos E.S., Salem R. (2025). Radiation Segmentectomy and Modified Radiation Lobectomy for Unresectable Early-Stage Intrahepatic Cholangiocarcinoma. J. Vasc. Interv. Radiol..

[31] Willowson K.P., Eslick E.M., Bailey D.L. (2021). Individualised dosimetry and safety of SIRT for intrahepatic cholangiocarcinoma. EJNMMI Phys..

[32] Mouli S., Memon K., Baker T., Benson A.B., Mulcahy M.F., Gupta R., Ryu R.K., Salem R., Lewandowski R.J. (2013). Yttrium-90 radioembolization for intrahepatic cholangiocarcinoma: Safety, response, and survival analysis. J. Vasc. Interv. Radiol..

[33] Schartz D.A., Porter M., Schartz E., Kallas J., Gupta A., Butani D., Cantos A. (2022). Transarterial Yttrium-90 Radioembolization for Unresectable Intrahepatic Cholangiocarcinoma: A Systematic Review and Meta-Analysis. J. Vasc. Interv. Radiol..

[34] Edeline J., Bridgewater J., Campillo-Gimenez B., Neveu E., Phelip J.-M., Neuzillet C., Boudjema K., Rolland Y., Valle J.W., Garin E. (2024). Chemotherapy with or without selective internal radiation therapy for intrahepatic cholangiocarcinoma: Data from clinical trials. Hepatology.

[35] Bourien H., Palard X., Rolland Y., Le Du F., Beuzit L., Uguen T., Le Sourd S., Pracht M., Manceau V., Lièvre A. (2019). Yttrium-90 glass microspheres radioembolization (RE) for biliary tract cancer: A large single-center experience. Eur. J. Nucl. Med. Mol. Imaging.

[36] Soydal C., Araz M., Demir B., Ones T., Sonmezoglu K., Selcuk N.A., Balli T., Isık E.G., Salancı B.V., Derebek E. (2025). Transarterial Radioembolization in the TACOME Trial: Dosimetric Analysis and Clinical Features in Predicting Response and Overall Survival. J. Nucl. Med..

[37] Alsultan A.A., van Roekel C., Barentsz M.W., Smits M.L.J., Kunnen B., Koopman M., Braat A.J., Bruijnen R.C., de Keizer B., Lam M.G. (2021). Dose-Response and Dose-Toxicity Relationships for Glass 90Y Radioembolization in Patients with Liver Metastases from Colorectal Cancer. J. Nucl. Med..

[38] Kurilova I., Bendet A., Fung E.K., Petre E.N., Humm J.L., Boas F.E., Crane C.H., Kemeny N., Kingham T.P., Cercek A. (2021). Radiation segmentectomy of hepatic metastases with Y-90 glass microspheres. Abdom. Radiol..

[39] Dimopoulos P.M., Sotirchos V.S., Dunne-Jaffe C.B., Petre E.N., Gonen M., Zhao K., Kirov A.S., Crane C., D’aNgelica M., Connell L.C. (2025). Voxel-Based Dosimetry Predicts Local Tumor Progression Post 90 Y Radiation Segmentectomy of Colorectal Liver Metastases. Clin. Nucl. Med..

[40] Dimopoulos M.-P., Sotirchos V.S., Dunne-Jaffe C., Mitchell A., Petre E.N., Alexander E.S., Gonen M., Rao D., Connell L.C., Soares K. (2025). Tumor Absorbed Dose Predicts Survival and Local Tumor Control in Colorectal Liver Metastases Treated with 90Y Radioembolization. Cardiovasc. Interv. Radiol..

[41] Ebbers S.C., van Roekel C., Braat M.N.G.J.A., Barentsz M.W., Lam M.G.E.H., Braat A.J.A.T. (2022). Dose-response relationship after yttrium-90-radioembolization with glass microspheres in patients with neuroendocrine tumor liver metastases. Eur. J. Nucl. Med. Mol. Imaging.

[42] Chansanti O., Jahangiri Y., Matsui Y., Adachi A., Geeratikun Y., Kaufman J.A., Kolbeck K.J., Stevens J.S., Farsad K. (2017). Tumor Dose Response in Yttrium-90 Resin Microsphere Embolization for Neuroendocrine Liver Metastases: A Tumor-Specific Analysis with Dose Estimation Using SPECT-CT. J. Vasc. Interv. Radiol..

[43] Watanabe M., Leyser S., Theysohn J., Schaarschmidt B., Ludwig J., Fendler W.P., Moraitis A., Lahner H., Mathew A., Herrmann K. (2024). Dose-Response Relationship in Patients with Liver Metastases from Neuroendocrine Neoplasms Undergoing Radioembolization with 90Y Glass Microspheres. J. Nucl. Med..

[44] Gordon A.C., Savoor R., Kircher S.M., Kalyan A., Benson A.B., Hohlastos E., Desai K.R., Sato K., Salem R., Lewandowski R.J. (2025). Yttrium-90 Radiation Segmentectomy for Treatment of Neuroendocrine Liver Metastases. J. Vasc. Interv. Radiol..

[45] Ridouani F., Soliman M.M., England R.W., Hsu M., Moskowitz C.S., Doustaly R., Sofocleous C.T., Boas F.E., Yarmohammadi H., Deipolyi A.R. (2021). Relationship of radiation dose to efficacy of radioembolization of liver metastasis from breast cancer. Eur. J. Radiol..

[46] Demir B., Soydal C., Kucuk N.O., Celebioglu E.C., Bilgic M.S., Oz D.K., Elhan A.H., Kir K.M. (2024). Voxel-based dosimetry with integrated Y-90 PET/MRI and prediction of response of primary and metastatic liver tumors to radioembolization with Y-90 glass microspheres. Ann. Nucl. Med..

[47] Sandow T., Gimenez J., Nunez K., Tramel R., Gilbert P., Oliver B., Cline M., Fowers K., Cohen A., Thevenot P. (2024). Using Voxel-Based Dosimetry to Evaluate Sphere Concentration and Tumor Dose in Hepatocellular Carcinoma Treated with Yttrium-90 Radiation Segmentectomy with Glass Microspheres. J. Vasc. Interv. Radiol..

[48] Pasciak A.S., Abiola G., Liddell R.P., Crookston N., Besharati S., Donahue D., Thompson R.E., Frey E., Anders R.A., Dreher M.R. (2020). The number of microspheres in Y90 radioembolization directly affects normal tissue radiation exposure. Eur. J. Nucl. Med. Mol. Imaging.

[49] Pasciak A.S., Bourgeois A.C., Bradley Y.C. (2016). A Microdosimetric Analysis of Absorbed Dose to Tumor as a Function of Number of Microspheres per Unit Volume in 90Y Radioembolization. J. Nucl. Med..

[50] Maxwell A.W.P., Mendoza H.G., Sellitti M.J., Camacho J.C., Deipolyi A.R., Ziv E., Sofocleous C.T., Yarmohammadi H., Maybody M., Humm J.L. (2022). Correction to: Optimizing ^90^Y Particle Density Improves Outcomes After Radioembolization. Cardiovasc. Interv. Radiol..

[51] Ahmad Y., Asad N., Ahmad R., Reed W., Ahmed O. (2023). Geospatial and Socioeconomic Disparities in Access to Interventional Radiology Care in the United States. J. Vasc. Interv. Radiol..

[52] Emmons E.C., Bishay S., Du L., Krebs H., Gandhi R.T., Collins Z.S., O’hAra R., Akhter N.M., Wang E.A., Grilli C. (2022). Survival and Toxicities after ^90^Y Transarterial Radioembolization of Metastatic Colorectal Cancer in the RESIN Registry. Radiology.

[53] Thomas M.A., Mahvash A., Abdelsalam M., Kaseb A.O., Kappadath S.C. (2020). Planning dosimetry for 90 Y radioembolization with glass microspheres: Evaluating the fidelity of 99m Tc-MAA and partition model predictions. Med. Phys..

